# Interleukin-1β Inhibits Ovarian Cancer Cell Proliferation and Metastasis Through the MAPK/MMP12 Pathway

**DOI:** 10.3390/ijms26073287

**Published:** 2025-04-01

**Authors:** Zhenling Ma, Jiajia Zhang, Zhenzhen Li, Yiyang Zhu, Xulu Han, Lanxiang Lei, Kun Cheng, Wei Liu

**Affiliations:** College of Life Sciences, Henan Agricultural University, Zhengzhou 450002, China; xmzl@henau.edu.cn (Z.M.); m144187@163.com (J.Z.); li3428331904@163.com (Z.L.); 19503809359@163.com (Y.Z.); han12282025@163.com (X.H.); 15137572918@163.com (L.L.); chengkun881111@foxmail.com (K.C.)

**Keywords:** epithelial ovarian cancer, interleukin-1β, tumor metastasis, matrix metalloproteinase-12

## Abstract

Epithelial ovarian cancer (EOC) is a gynecological tumor with high mortality. Despite aggressive treatment, survival rates for patients with advanced EOC are low, and more effective methods of diagnosis and treatment are urgently needed. Inflammation and cancer are strongly associated; however, the mechanisms that mediate this relationship are not fully understood. In this study, we found that the expression of interleukin-1β (IL-1β), a proinflammatory cytokine, increased in an ovarian cancer tissue microarray (TMA) and inhibited A2780 and SKOV3 cell viability and metastasis. Recombinant IL-1β protein and the overexpression of IL-1β decreased the proliferation and metastasis of ovarian cancer cells. IL-1β deficiency promoted proliferation and metastasis. Moreover, transcriptome sequencing revealed that IL-1β downregulates the expression of matrix metalloproteinase 12 (MMP12). The signaling pathway involving MAPK/AP-1/MMP12 is involved in IL-1β-regulated ovarian cancer progression. Overall, we found that the proinflammatory cytokine IL-1β inhibits ovarian cancer cell viability and metastasis. These findings provided deeper insights into inflammation and cancer progression.

## 1. Introductions

Ovarian cancer (OC) is a gynecological tumor that ranks seventh among female malignancies [1]. There are three main types of carcinoma, which include sex-cord-stromal carcinoma, epithelial carcinoma, and germ-cell carcinoma. Among them, epithelial ovarian cancer (EOC) accounts for the highest proportion, up to 90%. There are five types of EOC, including high-grade serous carcinoma, low-grade serous carcinoma, endometrioid carcinoma, clear cell carcinoma, and mucinous carcinoma [2]. They have different clinical behaviors, treatment responses, and prognoses. In 2020, about 314,000 ovarian cancer patients were diagnosed, and about 207,000 ovarian cancer patients died. Under the assumption that the incidence remains unchanged at the 2020 level, experts predict that there could be about 428,000 new cases and 307,000 deaths per year in 2040 [3,4]. Owing to the limitations and ineffectiveness of existing screening methods, early detection is difficult. For this reason, nearly 70% of cases are found at an advanced stage [5]. The five-year survival rate for patients in the advanced stage is only 45.6%; thus, more effective ovarian cancer diagnostic techniques and drugs need to be developed [5].

Inflammation plays a key role in the development and treatment of tumors. Inflammatory cells and their secreted cytokines can regulate the survival, metastasis, and differentiation of various cells in the tumor microenvironment, including macrophages, fibroblasts, and tumor cells [6,7]. Many types of cancer originate from infection and inflammation, and the development of cancer can be regarded as a disorder of the body’s tissue repair ability [8]. However, the recruitment of inflammatory cells may also contribute to immune function of the host, thus inhibiting tumor growth [9,10]. Cancer and inflammation are closely related; however, the mechanism by which cytokine signaling mediates this relationship remain unresolved and need to be further investigated. Proinflammatory interleukin-1β (IL-1β) is the most characteristic member of the interleukin-1 cytokine family. It is produced in response to inflammatory signals and activates an immune response by binding to its receptor, IL-1 receptor 1 (IL-1R1) [11,12]. IL-1β may promote tumorigenesis and tumor proliferation through different molecular mechanisms. An increase in the expression of IL-1β levels is associated with tumor progression. IL-1β promotes the growth and migration of tumors by influencing the differentiation of epithelial cells, the secretion of angiogenic molecules, and the expression of adhesion molecules, thus increasing tumor angiogenesis [13,14,15,16]. Several studies have shown that many antitumor therapeutic techniques induce IL-1β production [17]. Although the primary role of IL-1β is to promote tumors, some studies suggest that it may also be involved in antitumor immune responses [18,19].

C-jun N-terminal kinase (JNK), a member of the mitogen-activated protein kinase (MAPK) signaling pathway, is involved in the onset and development of cancer. It activates c-Jun primarily by phosphorylating N-terminal serine residues. JNK plays a key role in primary tumor growth, the regulation of cancer stem cell population, and the promotion of invasion and metastasis, and is considered to be an attractive target for anticancer therapy [20]. Several ATP competitive inhibitors and ATP non-competitive inhibitors have been developed and applied in vitro [20,21]. JNK plays a key role in the autophagy of ovarian cancer cells. Activation of the JNK pathway can induce autophagy, thus mediating the death and apoptosis of tumors in the context of malnutrition [21]. Matrix metalloproteinase 12 (MMP12), also referred to as macrophage metalloelastase, is a member of the matrix metalloproteinase family. The MMP12 gene is located on chromosome 11q22.3. The MMP12 level can increase in various solid tumors, such as pancreatic, stomach, non-small cell lung, and liver cancers, and may be used as a new biomarker to predict the prognosis of different types of cancer [22,23,24]. In hepatocellular carcinoma (LIHC), the upregulation of MMP12 is associated with tumor growth and progression by promoting angiogenesis, ultimately leading to poorer patient outcomes [25]. In lung adenocarcinoma (LUAD), the MMP12 protein level in tumor tissue increases significantly [26,27]. Ovarian cancer patients with high levels of MMP12 mRNA have better overall survival [28]. Studies have shown that IL-1βcan induce JNK phosphorylation in osteoblast MC3T3-E1 cells and induce bone marrow mesenchymal stromal cell senescence through the JNK pathway [29,30]. However, the relationship between IL-1β, JNK, and MMP12 in ovarian cancer has not been reported.

This study revealed that the average urinary IL-1β in patients with ovarian benign and ovarian cancer is higher than that in healthy individuals [31]. Other studies have shown that IL-1β can decrease the protein level of p53 in cancer-associated fibroblasts (CAFs) [32]. However, the role of IL-1β in the function of ovarian cancer cells is unclear and needs to be determined. In this study, we assessed the function of IL-1β in EOC cells, providing new insights into the relationship between inflammation and cancer progression.

## 2. Results

### 2.1. IL-1β Expression Is Increased Abnormally in Ovarian Cancer Tissues and Cells

**Figure 1:**
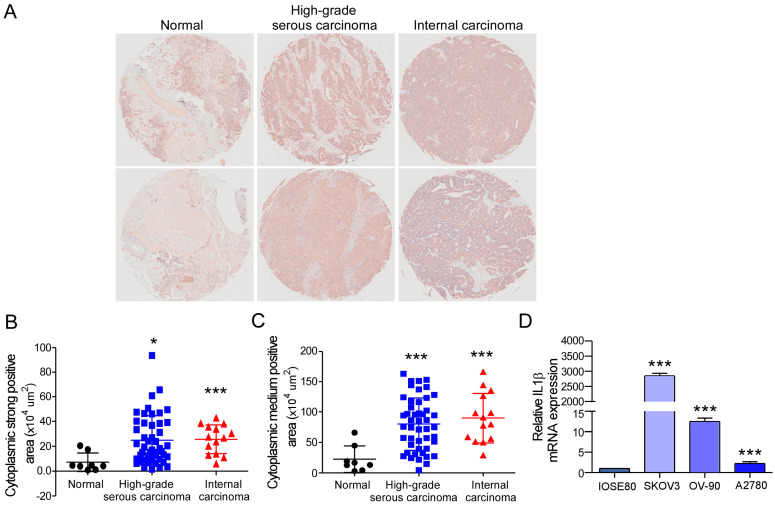
IL-1β is upregulated in ovarian cancer. Immunohistochemical staining of the ovarian cancer TMA with an anti-IL-1β antibody. The distribution of IL-1β in the cytoplasm was quantified. (**A**) Representative images of immunohistochemical staining for IL-1β in normal, high-grade serous carcinoma, and internal carcinoma samples (20×). (**B**) Cytoplasmic strong positive area of the immunohistochemistry-stained samples in the TMA. (**C**) Cytoplasmic medium positive area of the immunohistochemistry-stained samples in the TMA. (**D**) The level of expression of the IL-1β mRNA in a normal ovarian epithelial cell line and three ovarian cancer cell lines. Statistical significance is indicated as follows: * *p* < 0.05 and *** *p* < 0.001.

We first analyzed the IL-1β protein level in an ovarian cancer TMA, ZL-OVA961, from Super Biotek Company. ZL-OVA961 consists of eight normal ovarian tissue samples, 47 high-grade serous carcinoma samples, and 14 internal carcinoma samples. To detect IL-1β expression, immunohistochemical analysis was performed with anti-IL-1β antibodies. The IL-1β protein level was greater in ovarian cancer tissues, including high-grade serous carcinoma and internal carcinoma (Figure 1A). The dyeing depth can be divided into strong, medium, and weak positive areas and negative areas. Next, different stained areas were calculated, and histochemistry scores were assessed. High-grade serous carcinoma and internal carcinoma had greater strongly positive areas and moderately positive areas than normal ovarian tissue (Figure 1B,C). However, no differences were found in the weak positive area or negative area (Supplementary Figure S1A,B). Compared to those of normal tissue, the histochemistry scores of high-grade serous carcinoma and internal carcinoma were higher but not significantly different (*p* = 0.1701 and 0.081, respectively) (Supplementary Figure S1C). Moreover, the level of expression of IL-1β in ovarian cancer cells was determined. The results revealed that the IL-1β level was higher in the ovarian cancer cells compared to the control cells (Figure 1D). To summarize, these results indicated the significant upregulation of IL-1β in ovarian cancer.

### 2.2. Recombinant IL-1β Inhibits A2780 Cell Survival, Migration, and Invasion

**Figure 2:**
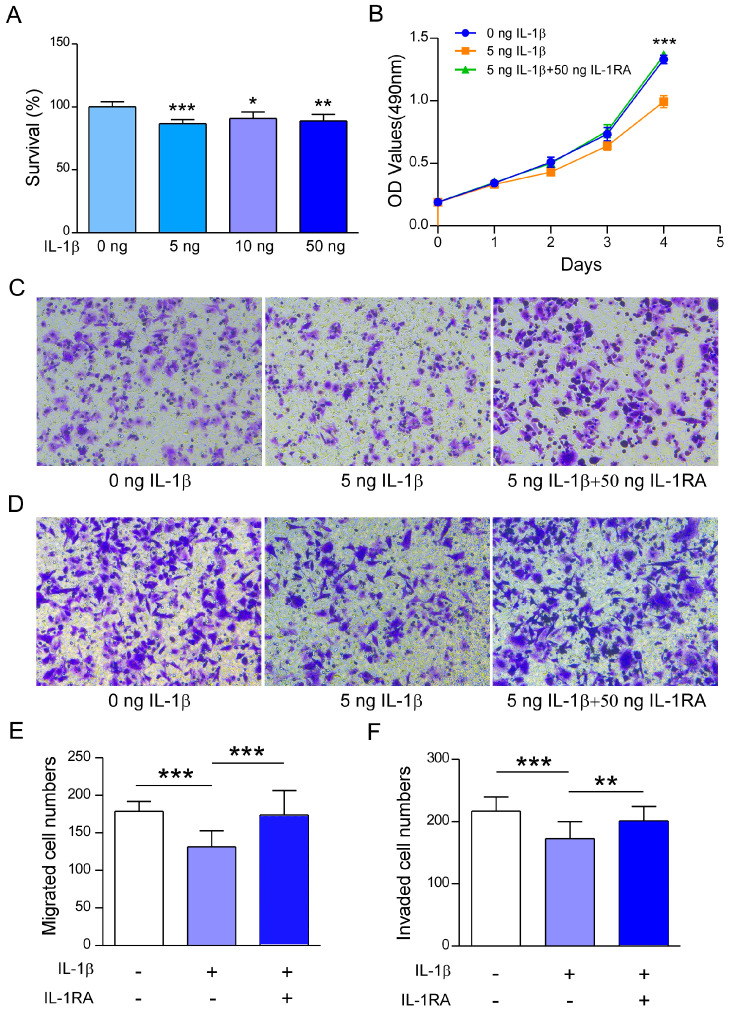
Inhibitory effects of IL-1β on the proliferation, migration, and invasion of A2780 cells. (**A**) The inhibitory effects of IL-1β on the viability of A2780 cells. A2780 cells were treated with 0, 5, 10, or 50 ng of IL-1β for 48 h, after which cell viability was measured by the MTT assay. (**B**) The proliferation of A2780 cells was inhibited by IL-1β. A2780 cells were treated with or without IL-1β and IL-1RA, and an MTT assay was subsequently conducted to assess cell proliferation. The inhibitory function of IL-1β was reversed by adding IL-1RA. (**C**) Representative micrographs of the migration of A2780 cells treated with IL-1β or IL-1β and IL-1RA (20×). (**D**) Representative micrographs of the invasion of A2780 cells treated with IL-1β or IL-1β and IL-1RA. (**E**) A graph illustrating the number of migrated cells. (**F**) A graph illustrating the number of invaded cells. Statistical significance is indicated as follows: * *p* < 0.05, ** *p* < 0.01, and *** *p* < 0.001.

The level of IL-1β increased significantly in the ovarian cancer TMA, which suggested that IL-1β may play a key role in ovarian cancer. To investigate the biological significance of IL-1β in ovarian cancer, recombinant IL-1β protein was used to detect its effect on the functions of ovarian cancer cells. To understand the effect of IL-1β on A2780 cell activity, an MTT experiment was conducted under stimulation with different concentrations of recombinant IL-1β. A cell viability assay revealed that recombinant IL-1β inhibited the survival rate of A2780 cells at concentrations ranging from 5 to 50 ng/mL (Figure 2A). Additionally, a concentration of 5 ng/mL was selected to conduct subsequent cell function experiments. A2780 cells were stimulated with recombinant IL-1β for four consecutive days, and their proliferation decreased (Figure 2B). The inhibitory effect was restored when the IL-1 receptor antagonist IL-1RA was added (Figure 2B).

Subsequently, transwell assays were conducted to study the role of recombinant IL-1β in the metastasis of ovarian cancer. The results suggested that the rate of cell migration was significantly lower in the recombinant IL-1β-treated A2780 cells than in the untreated A2780 cells (Figure 2C,E). Additionally, simultaneous treatment of A2780 cells with IL-1RA abolished the suppressive effect of recombinant IL-1β (Figure 2C,E). Moreover, recombinant IL-1β treatment inhibited the invasion ability of A2780 cells. Moreover, IL-1RA can counteract the inhibitory effect of recombinant IL-1β on the invasion of OC cells (Figure 2D,F). The above experiments revealed that recombinant IL-1β significantly reduced the survival and metastasis of ovarian cancer cells.

### 2.3. IL-1β Overexpression Is Related to a Reduction in Ovarian Cancer Cell Survival and Metastasis

**Figure 3:**
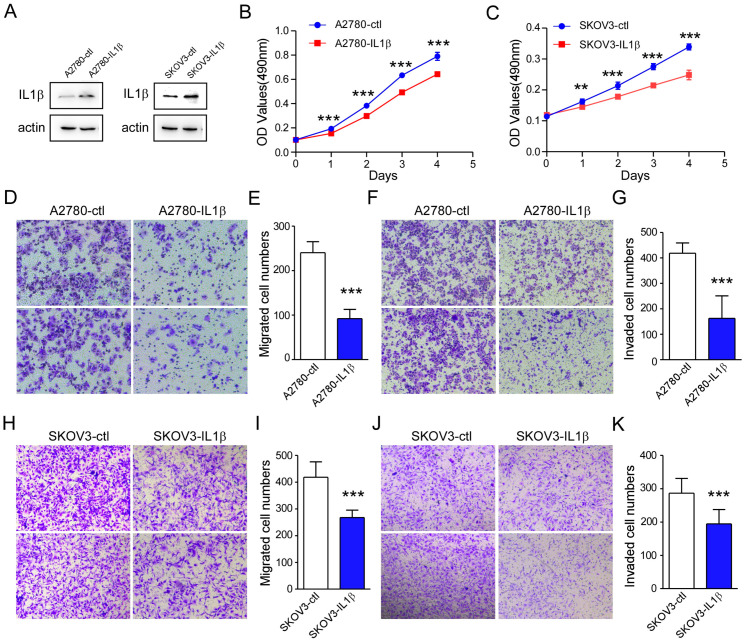
High expression of IL-1β suppresses ovarian cancer cell metastasis. (**A**) The levels of IL-1β in A2780 and SKOV3 cells increased after IL-1β was overexpressed. The protein levels of IL-1β and actin were detected by Western blotting analysis in A2780, IL-1β-overexpressing A2780, SKOV3, and IL-1β-overexpressing SKOV3 cells. (**B**) IL-1β overexpression decreased the proliferation of A2780 cells. (**C**) IL-1β overexpression decreased the proliferation of SKOV3 cells. (**D**) Representative photomicrographs of migrated A2780-ctl and A2780-IL-1β cells (20×). (**E**) The bar graphs show the mean ± SD of the number of migrated cells. (**F**) Representative photomicrographs of invading A2780-ctl and A2780-IL-1β cells. (**G**) Error bar graphs showing the mean numbers of invaded cells in the different groups. (**H**) Representative photomicrographs of migrated SKOV3-ctl and SKOV3-IL-1β cells. (**I**) The bar graphs show mean ± SD of the number of migrated cells. (**J**) Representative photomicrographs of invaded SKOV3-ctl and SKOV3-IL-1β cells. (**K**) Error bar graphs show the mean number of invaded cells in the different groups. Each experiment was repeated 3 times and 2 representative pictures were selected. Statistical significance is indicated as follows: ** *p* < 0.01, and *** *p* < 0.001.

Next, we determined whether IL-1β overexpression affects the function of ovarian cancer cells. First, stable IL-1β-overexpressing cell lines were produced using a lentiviral vector. Cells transfected with a blank vector were used as controls. The overexpression efficiency of IL-1β was subsequently verified by WB and qPCR assays. The results revealed that IL-1β was overexpressed in ovarian cancer cells (Figure 3A and Figure S2). A2780 and SKOV3 cells with high IL-1β expression exhibited a decrease in cell proliferation (Figure 3B,C). Furthermore, a cell migration assay indicated that the migration of IL-1β-overexpressing A2780 cells decreased significantly (Figure 3D,E). Similarly, the IL-1β-overexpressing A2780 cells presented a significant reduction in aggressiveness (Figure 3F,G). Similar results were found in IL-1β-overexpressing SKOV3 cells (Figure 3H–K). These findings indicated that the overexpression of IL-1β inhibits ovarian cancer cell survival and metastasis.

### 2.4. IL-1β Knockdown Promotes A2780 Cell Survival and Metastasis

**Figure 4:**
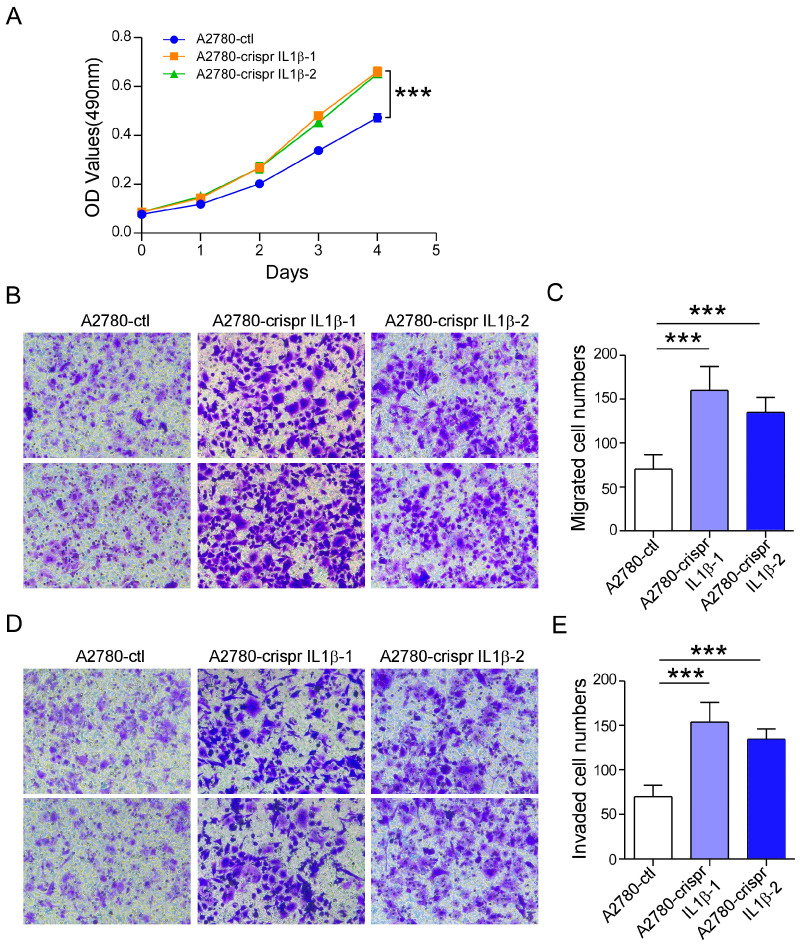
Knockdown of IL-1β promotes the function of ovarian cancer cells. (**A**) Knocking down of IL-1β promoted the proliferation of A2780 cells. (**B**) Representative micrographs of the migration of A2780-ctl, A2780-crispr-IL-1β-1, and A2780-crispr-IL-1β-2 cells (20×). (**C**) Error bar graphs show the mean number of migrated cells in the different groups. (**D**) Representative micrographs of the invasion of A2780-ctl, A2780-crispr-IL-1β-1, and A2780-crispr-IL-1β-2 cells. (**E**) Error bar graphs show the mean number of invaded cells in the different groups. Statistical significance is indicated as follows: *** *p* < 0.001.

We knocked down the expression of IL-1β using the CRISPR/Cas9 system. First, PX-459-sgIL-1β-1, PX-459-sgIL-1β-2, and PX-459-sgIL-1β-3 plasmids were constructed. Then, the recombinant plasmids were cotransfected into A2780 cells to construct A2780-crispr-IL-1β-1 and A2780-crispr-IL-1β-2 cells. Stable knockdown cells were subsequently prepared. After knocking down IL-1β, A2780-crispr IL-1β cells presented significantly lower expression of the IL-1β protein compared to control cells, as shown by the results of the Western blotting assays (Supplementary Figure S3). In the MTT assay, cell proliferation was significantly promoted in A2780-crispr IL-1β cells (Figure 4A). The number of migrating A2780-crispr IL-1β cells also increased significantly (Figure 4B,C). Similarly, an increase in cell invasion was found after IL-1β was silenced (Figure 4D,E). These results indicated that knocking down IL-1β contributes to ovarian cancer cell survival and metastasis.

### 2.5. Screening for Differentially Expressed Genes Regulated by IL-1β

**Figure 5:**
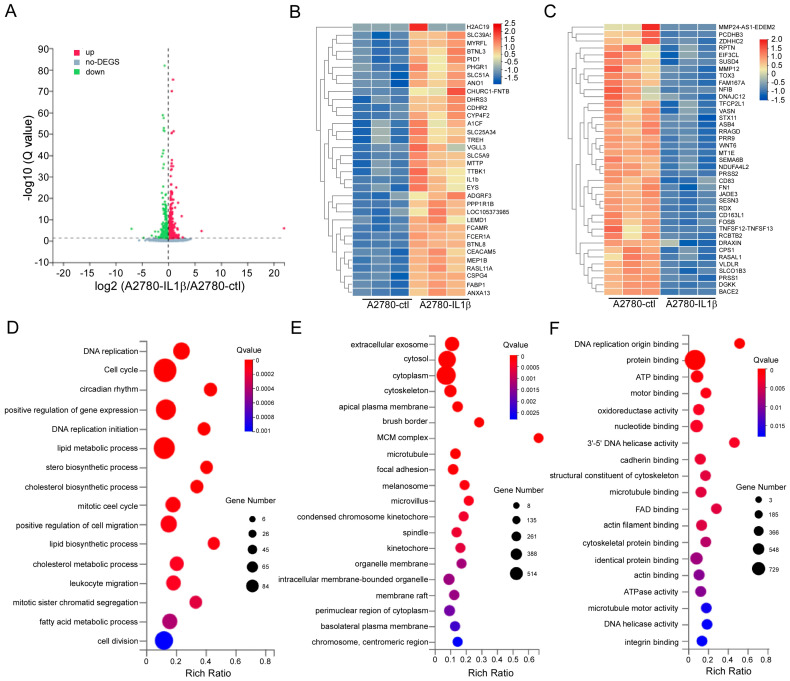
Screening for differentially expressed genes in IL-1β-overexpressing A2780 cells. A2780 and IL-1β-overexpressing A2780 cells were used to perform RNA sequencing, and the resulting transcriptomic changes were analyzed. (**A**) The volcano plot shows genes that were differentially expressed between A2780 and IL-1β-overexpressing A2780 cells, based on a |log2-fold fold change| > 1 and *p* < 0.05 (blue dots: significantly downregulated genes; red dots: significantly upregulated genes; gray dots: undifferentiated genes). DEG: differentially expressed gene. A heat map of upregulated genes (**B**) and downregulated genes (**C**) with log2 (fold change) ≥ 1.0. (**D**) The underlying biological process of the differentially expressed genes. (**E**) The underlying cellular component of the differentially expressed genes. (**F**) The underlying molecular functions of the differentially expressed genes.

To investigate the regulatory effects of IL-1β, RNA sequencing was conducted on A2780-ctl and IL-1β-overexpressing A2780 cells. First, the differentially expressed genes were analyzed. We found that 1117 genes were differentially expressed. The differentially expressed genes from A2780-ctl- and IL-1β-overexpressing A2780 cells are presented in the volcano plot (Figure 5A). Compared to the A2780-ctl group, 618 DEGs were upregulated, and 499 were downregulated. Based on |log2 fold change| > 1 and *p* < 0.05, the expression of 73 genes was significantly different; among them, 34 genes were upregulated and 39 genes were downregulated (Figure 5B,C). To understand their function, GO analysis was performed, which provided insights into the underlying biological processes, cellular components, and molecular functions of IL-1β that affect the progression of ovarian cancer. The significantly enriched biological processes included the cell cycle, the positive regulation of gene expression, cell division, and the lipid metabolic processes (Figure 5D). The cellular components were related mainly to the cytoplasm, cytosol, and extracellular exosome (Figure 5E). The molecular functions included protein binding, nucleotide binding, and ATP binding (Figure 5F).

### 2.6. IL-1β Regulates MMP12 Through the MAPK/AP-1 Signaling Pathway, and Reduced MMP12 Suppresses A2780 Cell Proliferation and Metastasis

**Figure 6:**
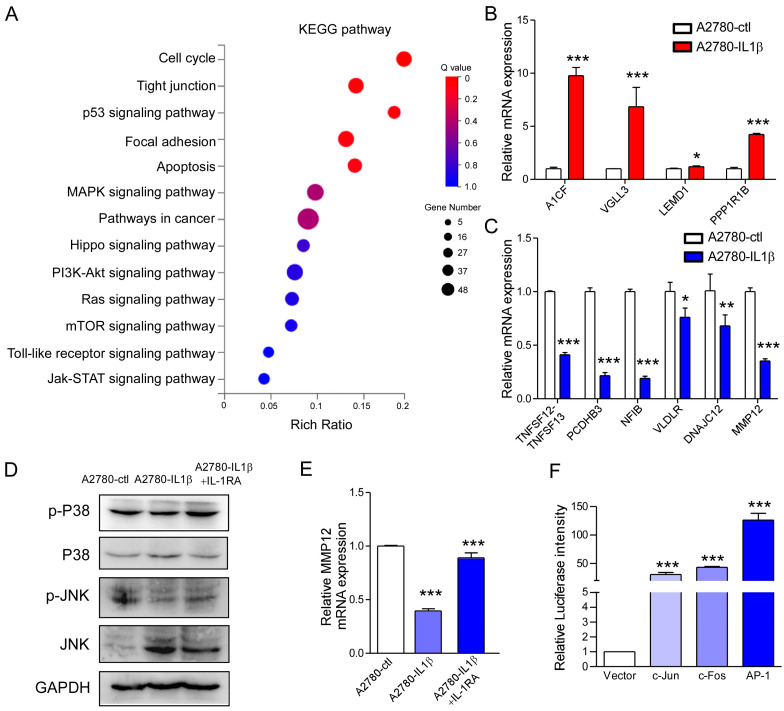
IL-1β regulates MMP12 expression through the MAPK/AP-1 pathway. (**A**) KEGG functional analysis was conducted to identify the enriched signaling pathways affected by IL-1β. Four upregulated and six downregulated genes were confirmed by conducting qPCR assay. (**B**) Quantitative PCR analysis was conducted to determine the expression levels of A1CF, VGLL3, LEMD1, and PPP1R1B. (**C**) Quantitative PCR analysis was conducted to determine the expression levels of TNFSF12-TNFSF13, PCDHB3, NFIB, VLDLR, DNAJC12, and MMP12. (**D**) The levels of phosphorylated P38 and JNK decreased in IL-1β-overexpressing A2780 cells. This downregulation was reversed by adding IL-1RA. The protein levels of total or phosphorylated P38 and JNK were determined by Western blotting analysis. GAPDH was used for endogenous normalization. (**E**) Quantitative PCR analysis was conducted to determine the expression level of MMP12 in IL-1β-overexpressing A2780 cells treated with or without IL-1RA. (**F**) Luciferase reporter analysis was conducted to assess MMP12 promoter activity regulated by c-Jun, c-Fos, and AP-1. * *p* < 0.05, ** *p* < 0.01, and *** *p* < 0.001 indicate significance.

To elucidate the underlying mechanisms by which IL-1β hinders the biological function of ovarian cancer, the Kyoto Encyclopedia of Genes and Genomes (KEGG) analysis was conducted. The results showed that the differentially expressed genes were related to the cell cycle, tight junctions, the p53 signaling pathway, the PI3K-Akt signaling pathway, focal adhesion, the toll-like receptor signaling pathway, apoptosis, the MAPK signaling pathway, pathways related to cancer, the hippo signaling pathway, the ras signaling pathway, the mTOR signaling pathway, and the Jak-STAT signaling pathway (Figure 6A). Additionally, we selected four genes whose expression was most significantly upregulated and six genes whose expression was most significantly downregulated (Table 1). The four upregulated genes included LEM domain containing 1, Apobec-1 complementation factor, vestigial-like family member 3, and protein phosphatase 1 regulatory inhibitor subunit 1B. The six downregulated genes included tumor necrosis factor superfamily members, protocadgerin beta 3, nuclear factor 1B, very low-density lipoprotein receptor, DnaJ heat shock protein family (HSP40) member C12, and matrix metallopeptidase 12. The mRNA levels of these 10 genes were subsequently detected by qPCR analysis. The change trend of these genes was consistent with the transcriptome data (Figure 6B,C). Some studies have suggested that the expression of MMP12 increases in various of cancers. It is closely associated with poor prognosis in many cancers [33]. Therefore, we selected MMP12 for subsequent verification experiments. To determine how IL-1β regulates the expression of MMP12, we evaluated the phosphorylation of kinases related to the MAPK, Akt, and NF-κB pathways by Western blotting analysis. The results indicated that the phosphorylation of P38 and JNK was greater in the IL-1β-overexpressing A2780 cells than in the control cells (line 2 of Figure 6D). Pretreatment with the IL-1 receptor antagonist IL-1RA reversed the increase in P38 and JNK phosphorylation (line 3 of Figure 6D). However, phosphorylated NF-κB and Akt showed the opposite trend (Supplementary Figure S4). In IL-1β-overexpressing A2780 cells, the mRNA level of MMP12 decreased, whereas it increased after IL-1RA was added (Figure 6E). AP-1 is a dimer composed of c-Jun and c-Fos and a universal transcription factor for the P38 and JNK pathways. We subsequently examined whether AP-1 regulates MMP12 transcription by conducting a dual luciferase assay. The findings revealed that Ap-1 promoted the activity of the MMP12 promoter (Figure 6F). Moreover, the activity of the MMP12 was greater when jun and fos were added simultaneously.

**Figure 7:**
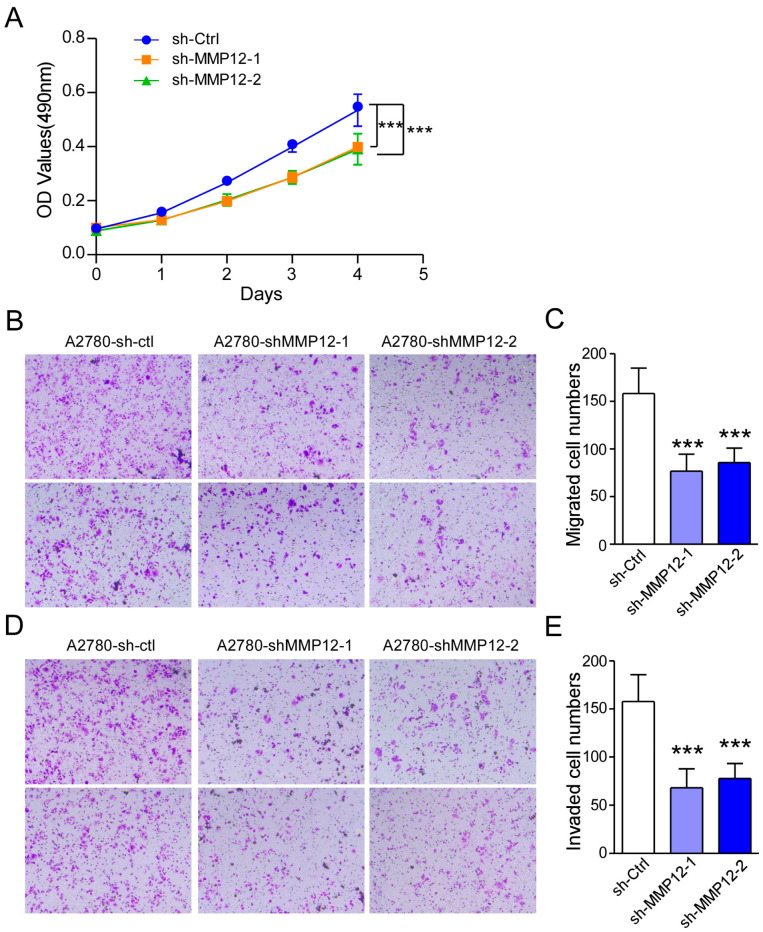
Silencing of MMP12 suppresses the proliferation, migration, and invasion of A2780 cells. (**A**) Knocking down MMP12 inhibited A2780 cell proliferation. (**B**) Representative micrographs of the migration of A2780-ctl, A2780-shMPP12-1, and A2780-shMPP12-2 cells (20×). (**C**) Representative micrographs of the invasion of A2780-ctl, A2780-shMPP12-1, and A2780-shMPP12-2 cells. (**D**) Error bar graphs show the mean number of migrated cells in the different groups (20×). (**E**) Error bar graphs show the mean number of invaded cells in the different groups. Statistical significance is indicated as follows: *** *p* < 0.001.

We hypothesized that MMP12 may be related to the IL-1β-regulated metastasis of OC. To test this hypothesis, an MMP12-knockdown A2780 cell line was constructed with a shRNA-MMP12 plasmid. The mRNA level of MMP12 in A2780-shMMP12 cells was reduced as determined by qPCR assays (Supplementary Figure S5). Next, an MTT experiment was conducted. The results revealed that the proliferation capacity of A2780-shMMP12 cells was reduced (Figure 7A). Moreover, we performed transwell migration and invasion assays. Compared to that of A2780-ctl cells, the migration of A2780-shMMP12 cells decreased (Figure 7B,C). Consistent with these findings, we detected reduced invasion in A2780-shMMP12 cells (Figure 7D,E). These findings suggested that IL-1β may inhibit the metastasis of ovarian cancer through the MAPK/AP-1/MMP12 signaling pathway.

## 3. Discussion

The relationship between inflammation and cancer is complex. Inflammatory cells play an important role in tumor progression. Inflammatory cells can promote tumor progression by expressing various cytokines [7,8]. Additionally, the host recruits tumor cells to resist tumor growth, thus inhibiting tumor growth [9,10]. The ovarian cancer microenvironment is a type of proinflammatory microenvironment. It is rich in tumor necrosis factor α (TNF-α), IL-1β, IL-6, and other chemokines and cytokines, which stimulate cancer cell growth, promote the formation of blood vessels, and reshape the extracellular matrix [34]. These factors may be secreted by tumor cells or produced by immune cells. Among these factors, IL-1β plays a contradictory role in tumor development. IL-1β can promote and inhibit tumor progression [13,14,18,19]. On the one hand, it can promote endothelial cell activation and angiogenesis; on the other hand, it induces an antigen-specific T lymphocyte response. IL-1β promotes the growth and migration of tumors by influencing the differentiation of epithelial cells, the secretion of angiogenic molecules, and the expression of adhesion molecules, thus increasing tumor angiogenesis [13,14,15,16]. IL-1β has been shown to play a protective role in chemically induced colon cancer mouse models [35]. In myeloma studies, IL-1a and IL-1β have been shown to work synergically with IFN-γ to induce tumor-killing activity in tumor-infiltrating macrophages [36,37]. Some studies have shown that IL-1β mRNA and protein levels are greater in patients with gastric esophageal carcinoma and squamous cell carcinoma than in controls. The expression of high levels of IL-1β was associated with lower overall survival and progression-free survival in patients with non-small cell lung cancer (NSCLC) and pancreatic cancer who were administered chemotherapy [38,39]. Quantitative analysis of IL-1β in cervical cancer tissue by qPCR indicated that women with low levels of IL-1β were at higher risk [40]. Conversely, concerning breast cancer, patients with high IL-1β mRNA had a better prognosis than those with low expression [41]. In nasopharyngeal carcinoma, immunohistochemical analysis revealed that patients with upregulated IL-1β expression had better local recurrence-free and disease-free survival [42]. This increase in IL-1β levels did not reflect IL-1β activity.

Studies have indicated that the plasma levels of IL-6, TNF-α, IL-1β, and other cytokines in patients with EOC are higher than those in normal controls [43]. The Urine IL-1β level increases in samples from benign and OC patients compared to healthy individuals [31]. The level of IL-1β in the ascites or peritoneal fluid of OC patients was significantly greater than that in the control group, and higher IL-1β in ascites was significantly associated with poor histopathological grade. Additionally, high levels of IL-1β in ascites and serum significantly reduce progression-free survival in patients with OC [44]. We first analyzed IL-1β expression in an ovarian cancer TMA consisting of eight normal ovarian tissue samples, 47 high-grade serous carcinoma samples, and 14 internal carcinoma samples. The IL-1β protein level was greater in ovarian cancer tissues, including high-grade serous carcinoma and internal carcinoma (Figure 1A). Moreover, the mRNA levels of IL-1β were greater in the three cancer cell lines than in the other ovarian epithelial cell lines (Figure 1D).

The matrix metalloproteinase MMP8 is associated with cancer progression, and IL-1β can induce MMP8 expression in 2780 ovarian cancer cell lines [45]. Schauer et al. reported that IL-1β, a chemokine secreted by ovarian cancer cells that acts on stromal cells, can inhibit the expression of p53 in CAFs [32]. Downregulating the expression of p53 in CAFs can increase the expression of the growth-regulating oncogenes α and IL-8 and other cytokines. Downregulating the expression of p53 increases the size of xenograft ovarian cancer tumors in mice. The researchers found that CAFs are important targets for blocking inflammation in the tumor microenvironment and reducing tumor growth. We treated A2780 and SKOV3 cells with recombinant IL-1β protein and IL-1RA, which increased and decreased the IL-1β level in A2780 and SKOV3 cells, respectively, and showed that IL-1β can decrease cell proliferation and metastasis. The role of IL-1β in cancer is controversial, and its tumor promotion and tumor suppression aspects have been reported. Overall, IL-1β may function by affecting tumor-specific Th1 cells, macrophages, or tumor cells in the tumor microenvironment. Our study only explored the effects of IL-1β on ovarian cancer cell lines, and its effects on tumor-associated macrophages and fibroblasts, as well as in vivo experiments and clinical analyses, need to be further studied.

During ovarian neogenesis and intraperitoneal metastasis, tumor cells need to break through the basement membrane. The key feature of MMPs is their ability to mediate the degradation of the basement membrane. The levels of these genes, such as MMP2, MMP10, and MMP25, are elevated in OC and can be used as prognostic biomarkers for OC [28]. In this study, we constructed an A2780 cell line in which MMP12 expression was silenced using shRNA. The results showed that A2780-shMMP12 cells exhibited inferior proliferation, migration, and invasion capabilities, suggesting an important function of MMP12 in the development of OC.

## 4. Materials and Methods

### 4.1. Cell Culture

Ovarian cancer (OC) cell lines, including A2780, OV-90, and SKOV3, were cultured in DMEM (HyClone) or McCoy’s 5a medium (HyClone). The normal epithelial cell line IOSE80 and 293T cell line were cultured in RPMI-1640 (HyClone) and DMEM, respectively. All media were supplemented with 10% fetal bovine serum (FBS; Biological Industries) and 100 units/mL penicillin-streptomycin (Cytiva). SKOV3 cells were purchased from the Chinese Academy of Sciences Cell Bank/Stem Cell Bank. A2780, OV-90, IOSE80, and 293T cells were preserved in our laboratory. All cells were cultured in a humidified atmosphere at 37 °C under 5% CO_2_.

### 4.2. Immunohistochemistry of the Cancer Tissue Microarray

The tissue microarray (TMA) ZL-OVA961 was purchased from Super Biotek Company and analyzed. This TMA contained eight normal ovarian tissue samples, 47 high-grade serous carcinoma samples, and 14 internal carcinoma samples. The expression of IL-1β was analyzed after TMA scanning. The region of interest was segmented according to the intensity of staining, and the staining area was calculated. The strong positive, medium positive, weak positive, and negative areas were subsequently calculated. The histochemistry score (HS) was obtained based on the staining intensity and number of positive cells. The larger the HS, the stronger the comprehensive positive intensity, i.e., the greater the level of expression of IL-1β.

### 4.3. Recombinant IL-1β and IL-1RA Treatment

Initially, A2780 cells were cultured in 6 cm dishes after being treated with serum-free DMEM overnight. The cells were stimulated with or without different concentrations (5, 10, or 50 ng/mL) of recombinant IL-1β protein for 48 h. To assess the effects of the IL-1R antagonist IL-1RA on the activity of ovarian cancer cells, 5 ng of IL-1RA was added. A2780 cells were assigned to three groups, including the 0 ng IL-1β group, the 5 ng IL-1β group, and the 5 ng IL-1RA group. Cell activity was measured by conducting the MTT assay.

### 4.4. MTT Assay

Cell viability and cell proliferation were measured by conducting MTT experiments. First, 2000 cells were seeded in a 96-well plate and cultured continuously for four days. Next, the MTT (Beyotime Biotechnology, Shanghai, China) solution was added, and the mixture was incubated for 3 h. Subsequently, dimethyl sulfoxide (DMSO) was added. Finally, the OD490 value was measured using a universal microplate reader. The cell viability was calculated from the optical density value. Each sample was analyzed in five wells.

### 4.5. Transwell Assay

Transwell assays were conducted following a method, described in another study [46]. After the cell were counted, they were seeded into the upper chambers of 8 μm pore (NEST) in 24-well plates. Then, 700 µL of complete medium was added to the lower chamber. The cells were cultured at 37 °C and 5% CO_2_ for 48 h. The chamber was removed from the incubator, and the cells from the upper chamber were removed with a cotton swab. The migrated cells that adhered to the lower side were fixed with 4% formaldehyde (Biosharp Life Sciences, Beijing, China) for 15 min and stained with 10% Giemsa stain (Biosharp Life Sciences, Beijing, China) for 30 min. Then, the migrated and invaded cells were counted and photographed under a light microscope. To conduct the cell invasion experiment, Matrigel (Corning) was added to coat the upper chambers.

### 4.6. Stable IL-1β-Overexpression and IL-1β-Knockdown Cells

The human IL-1β gene was inserted into the pCDH-CopGFP lentiviral plasmid. Human 293T cells were maintained in complete DMEM and cultured in a 10 cm dish. When the cells reached 70–80% confluence, pMDL, VSVG, and recombinant pCDH-IL-1β were cotransfected into the cells using Lipofectamine 8000 (Beyotime Biotechnology, Shanghai, China). After 48 h, the supernatants were harvested. A2780 and SKOV3 cells were subsequently incubated with the lentivirus-containing supernatants for 24 h. Positive cells were screened with puromycin. The IL-1β level in stable IL-1β-overexpressing cell lines was analyzed by qPCR and WB assays.

To construct IL-1β-knockdown cell lines, the sequences of the IL-1β sgRNAs were cloned and inserted into the pSpCas9 (BB)-2A-puro (PX459) plasmid. The primer sequences of the IL-1β sgRNAs are listed in Supplementary Table S1. The three recombinant vectors were denoted as PX-459-sgIL-1β-1, PX-459-sgIL-1β-2, and PX-459-sgIL-1β-3. The recombinant plasmids were cotransfected into A2780 cells to construct A2780-crispr-IL-1β-1 and A2780-crispr-IL-1β-2 cells. Untransfected cells did not survive in complete DMEM containing puromycin. The specific experimental methods were performed as described [46].

### 4.7. MMP12-Knockdown Cells

The sequences of MMP12 short hairpin RNA (shRNA) were inserted into the pLKO.1 plasmid and *Age*I and *Eco*RI were used to construct the pLKO.1-shMMP12 plasmid, which was transfected into A2780 cells using Lipofectamine 8000. The shRNAs of MMP12 are listed in Supplementary Table S1.

### 4.8. RNA Sequencing Analysis

First, A2780 and IL-1β-overexpressing A2780 cells were cultured. The cells were rinsed with cold PBS twice, after which total RNA was extracted. The samples were sent to the Beijing Genomics Institution for RNA sequencing. The sequencing data were subsequently mapped to the human reference genome (GRCh38). In pre-processing, low-quality reads were removed and batch effect was reducted. Differentially expressed genes were identified using the R package DEGseq (version 1.10.1). In this study, genes with |log2 (fold change)| ≥ 1 and *p* ≤ 0.05 were considered to be differentially expressed. To identify critical pathways and functions, the Kyoto Encyclopedia of Genes and Genomes (KEGG) and GO analyses were conducted.

### 4.9. RNA Extraction and qRT-PCR

Following the instructions provided with the reagent, total RNA was extracted from the experimental and control cells with TRIzol reagent (Zoman Biotechnology, Beijing, China), and then, the RNA was reverse-transcribed into cDNA using the First Strand cDNA Synthesis Kit (Yeasen Biotechnology, Shanghai, China), following the manufacturer’s protocols. Next, qPCR was subsequently performed with 2×HQ SYBR qPCR Mix (Zoman Biotechnology, Beijing, China) on an ABI Prism 7500 Sequence Detection System. The PCR conditions were as follows: initial denaturation at 94 °C for 5 min, followed by denaturation at 95 °C for 30 s, annealing at 58 °C for 30 s, extension at 72 °C for 20 s, and finally, after 40 cycles, a final extension at 72 °C for 10 min. GAPDH was used as a control for normalization. The sequences of the primers used in this study are listed in Supplementary Table S2.

### 4.10. Western Blotting Analysis

The cells in the control group and treatment group were lysed with RIPA buffer (Biosharp Life Sciences, Beijing, China) containing phosphatase inhibitor (Roche) and phenylmethylsulfonyl fluoride (PMSF; Sangon Biotech, Shanghai, China). Total protein was quantified using the BCA method (Beyotime Biotechnology, Shanghai, China). The extracted total protein samples were added to a 10% separation gel and transferred onto polyvinylidene fluoride membranes (Millipore). The samples were incubated with 5% milk for 2 h and then incubated with primary antibodies. Antibodies against IL-1β (Abbkine), phospho-JNK (CST), JNK (CST), phospho-P38 (CST), P38 (CST), phospho-AKT (CST), phospho-P65 (CST), actin (Zoman Biotechnology, Beijing, China), and GAPDH (TransGen Biotech, Beijing, China) were diluted to a ratio of 1:5000. The bands were visualized based on automatic exposure using a Tanon 5200 series fluorescence image analysis system (Tanon Technology, Shanghai, China). Detailed procedures are described in a previously published paper [47].

### 4.11. Dual Luciferase Assay

Using the genomic DNA of 293T cells as a template, the 2000 bp MMP12 promoter sequence was amplified by PCR and then linked to pGL3-basic. The plasmids overexpressing c-Fos and c-Jun and the Renilla luciferase plasmid were preserved in the laboratory. Luciferase reporter plasmids were transfected into 293T cells cultured in 24-well plates using Liposome 8000 when the cells reached about 70% confluence. The c-Jun:c-Fos:pGL3-MMP12-promoter transfection ratio was 1:1:2, whereas the Renilla:pGL3-MMP12-promoter ratio was 1:50. Following the instructions provided with the Dual Luciferase Reporter Gene Assay Kit (Yeasen Biotechnology, Shanghai, China), the cells were first lysed with a lysate buffer, after which the firefly and Renilla luciferase activities were detected. Firefly luciferase activity was normalized to Renilla. Each experiment was repeated three times.

### 4.12. Statistical Analysis

All data were analyzed using GraphPad Prism 5.0 software. All results were presented as the mean ± standard deviation. Statistical differences between paired groups were determined by Student’s *t*-tests, while three or more groups were identified by analysis of variance (ANOVA). NS indicates no statistical significance; * *p* < 0.05, ** *p* < 0.01, and *** *p* < 0.001 indicate significance.

## 5. Conclusions

In this study, we investigated the role of IL-1β in the function of A2780 and SKOV3 cells and elucidated the underlying mechanism. The results revealed that IL-1β was upregulated in the ovarian TMA. Stable overexpression of IL-1β in ovarian cancer cells inhibited growth and metastasis, while its knockdown in A2780 cells promoted cell activity and metastasis. To investigate the mechanism underlying the anticancer effects of IL-1β, a novel signaling pathway involving MAPK/AP-1/MMP12 was identified by RNA-Seq analysis. However, further in vivo trials and clinical data are needed to confirm these results. These findings provided new opportunities for follow-up research on the development and progression of cancer.

## Figures and Tables

**Table 1:** Four upregulated and six downregulated genes were screened.

Gene ID	Gene Name	Description	Fold Change/log_2_(IL1β/ctl)
29974	A1CF	Apobec-1 Complementation Factor	2.71
389136	VGLL3	Vestigial-like family member 3	1.93
93273	LEMD1	LEM domain containing 1	1.93
84752	PPP1R1B	Protein phosphatase 1 regulatory inhibitor subunit 1B	1.83
407977	TNFSF12-13	Tumor necrosis factor superfamily members	−6.99
56132	PCDHB3	Protocadgerin beta 3	−2.52
4781	NFIB	Nuclear factor IB	−2.38
7436	VLDLR	Very low density lipoprotein receptor	−1.94
56521	DNAJC12	DnaJ heat shock protein family (HSP40) member C12	−1.87
4321	MMP12	Matrix Metallopeptidase 12	−1.81

## Data Availability

All the data generated or analyzed during this study are included in this published article.

## Supplementary Materials

The following supporting information can be downloaded at: https://www.mdpi.com/article/10.3390/ijms26073287/s1.

## References

[1] Li Y., Li X.M., Yang K., Tong W.H. (2024). Advancements in ovarian cancer immunodiagnostics and therapeutics via phage display technology. Front. Immunol..

[2] Webb P.M., Jordan S.J. (2024). Global epidemiology of epithelial ovarian cancer. Nat. Rev. Clin. Oncol..

[3] Sung H., Ferlay J., Siegel R.L., Laversanne M., Soerjomataram I., Jemal A., Bray F. (2021). Global cancer statistics 2020: GLOBOCAN estimates of incidence and mortality worldwide for 36 cancers in 185 countries. CA Cancer J. Clin..

[4] Cabasag C.J., Fagan P.J., Ferlay J., Vignat J., Laversanne M., Liu L., Aa M.A., Bray F., Soerjomataram I. (2022). Ovarian cancer today and tomorrow: A global assessment by world region and Human Development Index using GLOBOCAN 2020. Int. J. Cancer.

[5] Chandra A., Pius C., Nabeel M., Nair M., Vishwanatha J.K., Ahmad S., Basha R. (2019). Ovarian cancer: Current status and strategies for improving therapeutic outcomes. Cancer Med..

[6] Elinav E., Nowarski R., Thaiss C.A., Hu B., Jin C., Flavell R.A. (2013). Inflammation-induced cancer: Crosstalk between tumours, immune cells and microorganisms. Nat. Rev. Cancer.

[7] Simon K., Teichmann S.A. (2024). Universal and tissue-specific fibroblasts in chronic inflammation and cancer. Cancer Cell.

[8] Coussens L.M., Werb Z. (2002). Inflammation and cancer. Nature.

[9] Erdman S.E., Poutahidis T. (2010). Cancer inflammation and regulatory T cells. Int. J. Cancer.

[10] Boccardi V., Marano L. (2024). Aging, cancer, and inflammation: The telomerase connection. Int. J. Mol. Sci..

[11] Bent R., Moll L., Grabbe S., Bros M. (2018). Interleukin-1 Beta-A friend or foe in malignancies?. Int. J. Mol. Sci..

[12] Yazdi A.S., Ghoreschi K. (2016). The interleukin-1 family. Adv. Exp. Med. Biol..

[13] Garlanda C., Dinarello C.A., Mantovani A. (2013). The interleukin-1 family: Back to the future. Immunity.

[14] Mantovani A., Barajon I., Garlanda C. (2018). IL-1 and IL-1 regulatory pathways in cancer progression and therapy. Immunol. Rev..

[15] Apte R.N., Krelin Y., Song X., Dotan S., Recih E., Elkabets M., Carmi Y., Dvorkin T., White R.M., Gayvoronsky L. (2006). Effects of micro-environment- and malignant cell-derived interleukin-1 in carcinogenesis, tumour invasiveness and tumour-host interactions. Eur. J. Cancer.

[16] Apte R.N., Dotan S., Elkabets M., White M.R., Reich E., Carmi Y., Song X., Dvozkin T., Krelin Y., Voronov E. (2006). The involvement of IL-1 in tumorigenesis, tumor invasiveness, metastasis and tumor-host interactions. Cancer Metastasis Rev..

[17] Hajek E., Krebs F., Bent R., Haas K., Bast A., Steinmetz I., Tuettenberg A., Grabbe S., Bros M. (2018). BRAF inhibitors stimulate inflammasome activation and interleukin 1 beta production in dendritic cells. Oncotarget.

[18] Rébé C., Ghiringhelli F. (2020). Interleukin-1beta and cancer. Cancers.

[19] Malkova A.M., Gubal A.R., Petrova A.L., Voronov E., Apte R.N., Semenov K.N., Sharoyko V.V. (2023). Pathogenetic role and clinical significance of interleukin-1β in cancer. Immunology.

[20] Latham S.L., O’Donnell Y.E.I., Croucher D.R. (2022). Non-kinase targeting of oncogenic c-Jun N-terminal kinase (JNK) signaling: The future of clinically viable cancer treatments. Biochem. Soc. Trans..

[21] Abdelrahman K.S., Hassan H.A., Abdel-Aziz S.A., Marzouk A.A., Narumi A., Konno H., Abdel-Aziz M. (2021). JNK signaling as a target for anticancer therapy. Pharmacol. Rep..

[22] Banerjee S., Lo W.C., Majumder P., Roy D., Ghorai M., Shaikh N.K., Kant N., Shekhawat M.S., Gadekar V.S., Ghosh S. (2022). Multiple roles for basement membrane proteins in cancer progression and EMT. Eur. J. Cell Biol..

[23] Marcos-Jubilar M., Orbe J., Roncal C., Machado F.J.D., Rodriguez J.A., Fernández-Montero A., Colina I., Rodil R., Pastrana J.C., Páramo J.A. (2021). Association of SDF1 and MMP12 with atherosclerosis and inflammation: Clinical and experimental study. Life.

[24] Guan P.P., Ding W.Y., Wang P. (2018). The roles of prostaglandin F2 in regulating the expression of matrix metalloproteinase-12 via an insulin growth factor-2-dependent mechanism in sheared chondrocytes. Signal Transduct. Target. Ther..

[25] Guo Z.Y., Jiang L.P. (2022). Matrix metalloproteinase 12 (MMP12) as an adverse prognostic biomarker of vascular invasion in hepatic cell carcinoma. Eur. Rev. Med. Pharmacol. Sci..

[26] Hung W.Y., Lee W.J., Cheng G.Z., Tsai C.H., Yang Y.C., Lai T.C., Chen J.Q., Chung C.L., Chang J.H., Chien M.H. (2021). Blocking MMP-12-modulated epithelial-mesenchymal transition by repurposing penfluridol restrains lung adenocarcinoma metastasis via uPA/uPAR/TGF-beta/Akt pathway. Cell Oncol..

[27] Lv F.Z., Wang J.L., Wu Y., Chen H.F., Shen X.Y. (2015). Knockdown of MMP12 inhibits the growth and invasion of lung adenocarcinoma cells. Int. J. Immunopathol. Pharmacol..

[28] Zeng L., Qian J., Zhu F., Wu F., Zhao H., Zhu H. (2020). The prognostic values of matrix metalloproteinases in ovarian cancer. J. Int. Med. Res..

[29] Noh J.M., Shen C., Kim S.J., Kim M.R., Kim S.H., Kim J.H., Park B.H., Park J.H. (2015). Interleukin-1β increases Angptl4 (FIAF) expression via the JNK signaling pathway in osteoblastic MC3T3-E1 cells. Exp. Clin. Endocrinol. Diabetes.

[30] Song M., Deng M., Peng Z., Dai F., Wang Y., Shu W., Zhou X., Zhang J., Hou Y., Yu B. (2024). Granulocyte colony-stimulating factor mediates bone loss via the activation of IL-1β/JNK signaling pathway in murine Staphylococcus aureus-induced osteomyelitis. Int. Immunopharmacol..

[31] Woolery K.T., Hoffman M.S., Kraft J., Nicosia S.V., Kumar A., Kruk P.A. (2014). Urinary interleukin-1β levels among gynecological patients. J. Ovarian Res..

[32] Schauer I.G., Zhang J., Xing Z., Guo X., Mercado-Uribe I., Sood A.K., Huang P., Liu J. (2013). Interleukin-1β promotes ovarian tumorigenesis through a p53/NF-κB-mediated inflammatory response in stromal fibroblasts. Neoplasia.

[33] Li G.S., Tang Y.X., Zhang W., Li J.D., Huang H.Q., Liu J., Fu Z.W., He R.Q., Kong J.L., Zhou H.F. (2024). MMP12 is a potential predictive and prognostic biomarker of various cancers including lung adenocarcinoma. Cancer Control.

[34] Macciò A., Madeddu C. (2012). Inflammation and ovarian cancer. Cytokine.

[35] Allen I.C., TeKippe E.M., Woodford R.M., Uronis J.M., Holl E.K., Rogers A.B., Herfarth H.H., Jobin C., Ting J.P. (2010). The NLRP3 inflammasome functions as a negative regulator of tumorigenesis during colitis-associated cancer. J. Exp. Med..

[36] Haabeth O.A., Lorvik K.B., Hammarstrom C., Donaldson I.M., Haraldsen G., Bogen B., Corthay A. (2011). Inflammation driven by tumour-specific Th1 cells protects against B-cell cancer. Nat. Commun..

[37] Haabeth O.A., Lorvik K.B., Yagita H., Bogen B., Corthay A. (2016). Interleukin-1 is required for cancer eradication mediated by tumor-specific Th1 cells. Oncoimmunology.

[38] McLoed A.G., Sherrill T.P., Cheng N.S., Han W., Saxon J.A., Gleaves L.A., Wu P., Polosukhin V.V., Karin M., Yull F.E. (2016). Neutrophil-Derived IL-1β impairs the efficacy of NF-κB inhibitors against lung cancer. Cell Rep..

[39] Kim J.W., Koh Y., Kim N.W., Ahn Y.O., Kim T.M., Han S.W., Oh Y., Lee S.H., Im S.A., Kim T.Y. (2013). Clinical implications of VEGF, TGF-beta1, and IL-1beta in patients with advanced non-small cell lung cancer. Cancer Res. Treat..

[40] Matamoros J.A., Silva M.I.F., Moura P.M.M.F., Leitão M.D.C.G., Coimbra E.C. (2019). Reduced expression of IL-1β and IL-18 proinflammatory interleukins increases the risk of developing cervical cancer. Asian Pac. J. Cancer Prev..

[41] Martínez-Reza I., Díaz L., Barrera D., Segovia-Mendoza M., Pedraza-Sánchez S., Soca-Chafre G., Larrea F., García-Becerra R. (2019). Calcitriol inhibits the proliferation of Triple-Negative breast cancer cells through a mechanism involving the proinflammatory cytokines IL-1β and TNF-α. J. Immunol. Res..

[42] Chen L.C., Wang L.J., Tsang N.M., Ojcius D.M., Chen C.C., Ouyang C.N., Hsueh C., Liang Y., Chang K.P., Chen C.C. (2012). Tumour inflammasome-derived IL-1β recruits neutrophils and improves local recurrence-free survival in EBV-induced nasopharyngeal carcinoma. EMBO Mol. Med..

[43] Santiago A.E., Paula S.O.C., Carvalho A.T., Cândido E.B., Furtado R., Filho A.L.S. (2023). Systemic inflammatory patterns in ovarian cancer patients: Analysis of cytokines, chemokines, and microparticles. Rev. Bras. Ginecol. Obstet..

[44] Mustea A., Pirvulescu C., Könsgen D., Braicu E.I., Yuan S., Sun P., Lichtenegger W., Sehouli J. (2008). Decreased IL-1 RA concentration in ascites is associated with a significant improvement in overall survival in ovarian cancer. Cytokine.

[45] Stadlmann S., Pollheimer J., Moser P., Raggi A., Amberger A., Margreiter R., Offner F., Mikuz G., Dirnhofer S., Moch H. (2003). Cytokine-regulated expression of collagenase-2 (MMP-8) is involved in the progression of ovarian cancer. Eur. J. Cancer..

[46] Liu W., Wang L., Zhang J., Cheng K., Zheng W., Ma Z. (2023). CC Chemokine 2 promotes ovarian cancer progression through the MEK/ERK/MAP3K19 signaling pathway. Int. J. Mol. Sci..

[47] Liu W., Yuan C., Fu B., Xie J., Li W., Zhang G., Ma Z., Jiao P. (2024). E3 ubiquitin ligase ANKIB1 attenuates antiviral immune responses by promoting K48-linked polyubiquitination of *MAVS*. Cell Rep..

