# Supplementary material for: Interleukin-1β Inhibits Ovarian Cancer Cell Proliferation and Metastasis Through the MAPK/MMP12 Pathway
Source: Int J Mol Sci. 2025 Apr 1;26(7):3287. doi: 10.3390/ijms26073287 (PMC11989259; doi:10.3390/ijms26073287)
Supplement: Supplementary file 1 [file ijms-26-03287-s001.zip › ijms-3495380-supplementary.pdf]

Supplementary Information

Supplementary Table S1 Primers for the construction of crispr IL-1β and shMMP12.

Primer name	Sequences
IL1β sgRNA-1	Fw CACCGGATGGCCCTAAACAGATGA
	Rv AAACTCATCTGTTTAGGGCCATCC
IL1β sgRNA-2	Fw CACCGATGGCCACAACAACTGACG
	Rv AAACCGTCAGTTGTTGTGGCCATC
IL1β sgRNA-3	Fw CACCGTTCGACACATGGGATAACG
	Rv AAACCGTTATCCCATGTGTCGAAC
shMMP12-1	GCCCGTATGGAGGAAACATTA
shMMP12-2	CTTGCTTGACTCTACTATTAA

Supplementary Table S2 Primers for qRT-PCR analyses

Primer name	Sequences
IL1β	Fw ATGATGGCTTATTACAGTGGCAA
	Rv GTCGGAGATTCGTAGCTGGA
A1CF	Fw TGTGGACAACTGCCGATTATTT
	Rv TGACATCGACAACACCTTCAGTA
VGLL3	Fw TATGGAGCGTCCCAGTATCTG
	Rv TGAATACCGCTAACTTCTTCTGC
LEMD1	Fw ATTGCAGAACCAACTTGAGAAGC
	Rv CGCGCAGTAGTCTCTCTCTT
PPP1R1B	Fw CAAGTCGAAGAGACCCAACCC
	Rv GCCTGGTTCTCATTCAAATTGCT
TNFSF12-13	Fw GTGTGGACGGGACAGTGAG
	Rv GCATCGGAACTCTGACAGTACAG
PCDHB3	Fw GGAGGAGAGCGATTTCTTAGACA
	Rv GAATAGCGTCTTGACTCGGAC
NFIB	Fw GCTGTGTCTTATCCAATCCCG
	Rv TGCCTTTGAACAGGATCACCA
VLDLR	Fw CTGGGTATGCGACGATGATG
	Rv CTTGGTGTGTATGACTGGCTG
DNAJC12	Fw AAGACTCATACCACCAAGATGGA
	Rv AATTTTGCGGGGAGACTGACT
MMP12	Fw CATGAACCGTGAGGATGTTGA
	Rv GCATGGGCTAGGATTCCACC

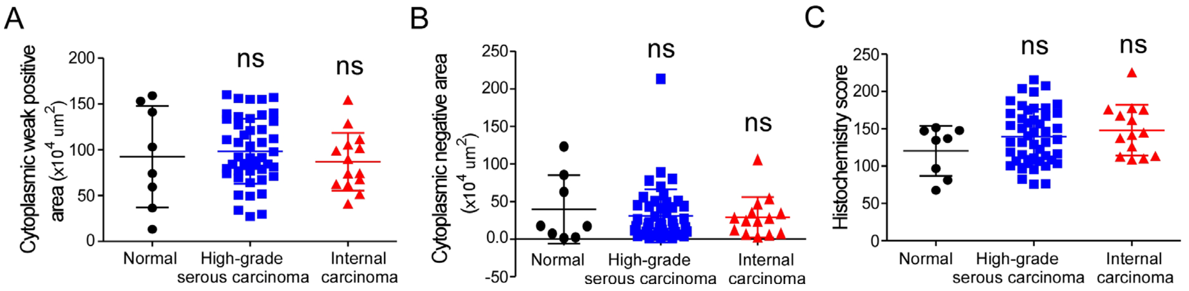

Supplementary Figure S1 (A) Cytoplasmic medium weak area of the immunohistochemistry-stained samples in the TMA. (B) Cytoplasmic medium negative area of the immunohistochemistry-stained samples in the TMA. (C) Histochemistry scores of the immunohistochemistry-stained samples in the TMA. Statistical significance is indicated as follows: NS indicates no statistical significance.

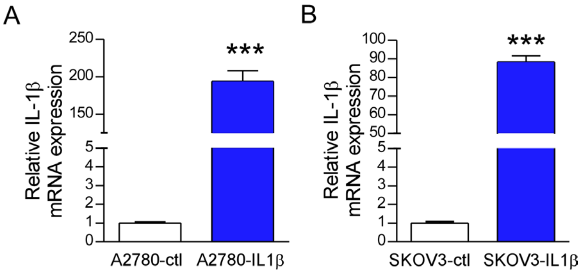

Supplementary Figure S2 Quantitative PCR analysis of IL-1β in IL-1β-overexpressing A2780 cells (A) and IL-1β-overexpressing SKOV3 cells (B). Statistical significance is indicated as follows: ***$p < 0.001$.

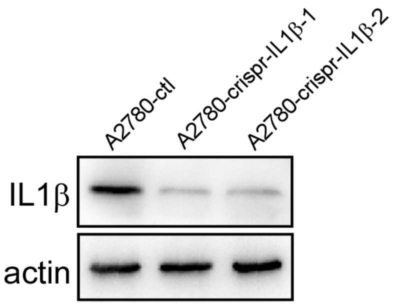

Supplementary Figure S3 The protein levels of IL-1β and actin were detected in A2780-ctl and A2780-crispr-IL-1β cells by Western blotting analysis.

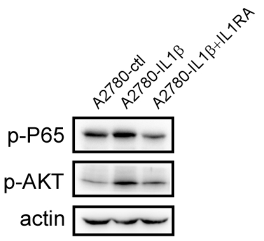

Supplementary Figure S4 The protein levels of phosphorylated P65 and AKT were detected by Western blotting analysist. Actin was used for endogenous normalization.

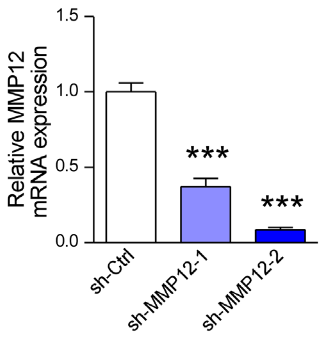

Supplementary Figure S5 Quantitative PCR analysis of MMP12 was performed in sh-MMP12 A2780 cells. Statistical significance is indicated as follows: ***$p < 0.001$.
